# Cultural competences among future nurses and midwives: a case of attitudes toward Jehovah’s witnesses’ stance on blood transfusion

**DOI:** 10.1186/s12909-024-05646-1

**Published:** 2024-06-15

**Authors:** Jan Domaradzki, Katarzyna Głodowska, Einat Doron, Natalia Markwitz-Grzyb, Piotr Jabkowski

**Affiliations:** 1 Department of Social Sciences and Humanities, Poznan University of Medical Sciences, Rokietnicka 7, Poznań, 60-806 Poland; 2 Independent researcher, Binyamina, Israel; 3 Faculty of Sociology, Adam Mickiewicz University, Poznań, Poland

**Keywords:** Blood transfusion, Cultural competences, Jehovah’s witnesses, Knowledge and attitudes, Midwifery students, Nursing students; transcultural nursing

## Abstract

**Background:**

Transcultural nursing recognises the significance of cultural backgrounds in providing patients with quality care. This study investigates the opinions of master’s students in nursing and midwifery regarding the attitudes of Jehovah’s Witnesses towards refusing blood transfusions.

**Methods:**

349 master’s students in nursing and midwifery participated in a quantitative study and were surveyed via the Web to evaluate their awareness of the stance of Jehovah’s Witnesses on blood transfusions and the ethical and legal dilemmas associated with caring for Jehovah’s Witness (JW) patients.

**Results:**

The study yielded three significant findings. It unequivocally demonstrates that nursing and midwifery students possess inadequate knowledge regarding Jehovah’s Witnesses’ stance on blood transfusions and their acceptance of specific blood products and medical procedures. Despite being cognisant of the ethical and legal dilemmas of caring for JW patients, students lack an understanding of patients’ autonomy to reject blood transfusions and their need for bloodless medicine. Students also articulated educational needs regarding cultural competencies regarding the Jehovah’s Witnesses’ beliefs on blood transfusions and non-blood management techniques.

**Conclusions:**

Healthcare professionals need the knowledge and skills necessary to provide holistic, patient-centred and culturally sensitive care. This study emphasises the urgent need for university curricula and nursing postgraduate training to include modules on transcultural nursing and strategies for minimising blood loss.

**Supplementary Information:**

The online version contains supplementary material available at 10.1186/s12909-024-05646-1.

## Background

Transcultural nursing entails nurses’ ability to approach each patient in a culturally sensitive and inclusive manner. It emphasises the need to consider patients’ cultural backgrounds, including values and norms, religious beliefs, traditional customs and lifestyles, as an essential part of quality care [1, 2], and has a central role in the healthcare domain, requiring nurses to embody cultural competence as an integral aspect of their daily practice [3]. The concept of cultural competence itself, which originated in social work, was developed in the 1970s by Madeleine Leininger, who emphasised that healthcare should include multiple aspects of culture, as they influence the way a person or a group perceives health and disease, approaches healthcare and copes with illness or death [4–6]. Cultural competence therefore entails a process that involves a heightened self-awareness, an appreciation of diversity and the acquisition of knowledge concerning cultural strengths [7]. Nurses conceptualise cultural competence as the capacity to understand cultural distinctions and the continuous process of effectively engaging with diverse individuals, helping them deliver quality care to a culturally diverse population [5, 6]. Culturally competent nurses show sensitivity to issues of culture, religion, race, ethnicity, gender and sexual orientation, highlighting their ability to communicate, perform cultural assessments and acquire knowledge related to diverse health practices.

Culturally competent nurses display a nuanced understanding of diverse cultural practices, enabling them to discern distinct patterns and formulate individualised care plans tailored to meet both healthcare goals and the individual needs of every patient [8]. While the overarching goal of transcultural nursing is to foster the values, knowledge and skills required for the provision of culturally different and sensitive care within a culturally diverse environment [2, 9], it is an integral part of holistic nursing which aims at addressing patients’ physical, psychological, emotional, spiritual and social needs, and underscores the imperative of individualised care [10]. In pursuing holistic care, nurses must meticulously consider cultural variations in their care plans, ensuring a thorough and culturally competent approach [8]. Appreciating patients’ cultural perspectives is paramount in delivering effective care and navigating intricate ethical scenarios, particularly within diverse cultural backgrounds [5, 6]. A detailed understanding of patients’ cultural backgrounds ensures a holistic and culturally competent approach to nursing care.

Given their prominence as the largest group of healthcare professionals, nurses are crucial in addressing global health challenges and disparities. The evolving landscape of global healthcare needs adjustments in nursing practice, positioning nurses at the forefront of addressing cultural backgrounds and global events that affect patients’ needs [11]. Nurses must be prepared to discern global healthcare issues and cultivate skills to attain cultural competences [12, 13].

While there are many groups of patients whose cultural background, religious beliefs or traditional customs are an essential part of their identity and may therefore influence their health and medical behaviour, psychological reaction to illness, treatment preferences and communication with the healthcare team, one notable example is Jehovah’s Witnesses (JWs), a Christian denomination founded in 1872 in the United States by Charles Taze Russell. Although JWs represent a religious minority in Poland, they have been enormously successful and, according to the Central Statistical Office, there are currently more than 114,000 JWs in Poland, making them the third largest religious group in Poland after Roman Catholics and Orthodox Christians [14].

One of the central beliefs adopted by JWs is their refusal to accept allogenic blood transfusions, even in cases in which the outcome may be death [15–21]. While JWs argue that there are also some medical grounds for refusing blood, this refusal is based on religious grounds and is the result of their interpretation of several verses in the Bible (*Genesis* 9:4; *Leviticus* 17:10; *Deuteronomy* 12:23; *Acts* 15:28–29) [22]. JWs therefore refuse transfusions of whole blood (including pre-operative autologous donation, i.e. auto-transfusion) and its four primary components (red cells, white cells, platelets and unfractionated plasma). In 2000, however, JWs were informed that ambiguity in the Bible meant that the use of blood fractions is not absolutely prohibited and that they may accept them as a matter of personal choice. JWs may consequently accept such derivatives of primary blood components as albumin solutions, cryoprecipitate, clotting factor concentrates and immunoglobulins [23, 24]. At the same time, although JWs reject allogeneic blood transfusions and pre-operative autologous transfusions, many other medical procedures are permitted and are left to the discretion of individual members, including blood donation, autologous transfusions, intra-operative blood salvage, dialysis, aphaeresis and cardiac bypass or organ transplants (on condition it is performed on a bloodless basis) [23, 24].

Many clinicians, including physicians (e.g. cardiac surgeons, obstetricians and anaesthesiologists), nurses and midwives who treat their patients with blood products [23–28], either whole blood transfusions or blood component therapy (e.g. red cell concentrates, fresh frozen plasma, platelet concentrates or cryoprecipitate) [29, 30] therefore face a challenging ethical and medico-legal dilemma: whether to respect patients’ autonomy and right to follow their religious beliefs, albeit this may result in death, or to remain faithful to the doctor’s duty to preserve life even against patients’ own wishes [16–19, 26, 31]. Although there is no official data, according to some estimates, up to one thousand JWs die in the United States each year due to their refusal of blood transfusions [32–34].

Since JWs carry a ready-made document regarding health care, a *No Blood* card, i.e. a declaration of the person’s resolution against blood transfusions, signed by the person and confirmed by two witnesses, which is compatible with the requirements of the Civil Code and is binding on an attending doctor. According to Polish law, any JW patients who have procedures performed on them that involve a blood transfusion against their will have the right to initiate any of three types of proceedings: criminal, civil or disciplinary [35–38].

If a JW patient refuses a blood transfusion, healthcare professionals must provide comprehensive information regarding treatment methods available with blood substitutes and other alternative methods used during surgical procedures. Since JWs reject pre-operative autologous blood donation, they may, for instance, be offered bloodless medical care, i.e. transfusion-free health care that uses neither allogeneic blood transfusion nor blood products during medical procedures and surgeries but instead uses blood conservation techniques and various blood transfusion alternatives, such as extra-corporeal circulation combined with recovery of patients’ blood from the surgical field in a closed circuit [39–41].

While earlier studies in Poland have focused on the moral (ethical) dilemmas and legal aspects of providing care to JW patients [35–38], there remains a shortage of research on the awareness among (future) healthcare professionals of JWs’ refusal of blood transfusions. This study, therefore, seeks to explore nursing and midwifery master’s students’ views on JWs’ attitudes towards blood transfusions, including (1) their awareness regarding JWs’ stance on blood transfusion, (2) students’ opinions on the ethical and legal dilemmas related to caring for JW patients, (3) students’ educational needs for non-blood management techniques, and (4) factors associated with future nurses’ and midwifes’ perception of JWs’ refusal of blood transfusion.

## Methods

### Study design

This research was part of a larger project aimed at assessing healthcare professionals’ attitudes towards JWs’ refusal of blood transfusions [42], but it was designed to explore the views of nursing and midwifery master’s students. It includes data from a self-administered, anonymised Web survey about future healthcare professionals’ awareness of JWs’ stance and the ethical and legal dilemmas related to their refusal of blood transfusions.

### Research tool

A modified version of a previously developed questionnaire that assessed the knowledge and attitudes of Polish nurses towards JWs’ stance in refusing blood transfusions was used [42]. The development of the questionnaire followed the guidelines of the European Statistical System [43]. It was constructed after the published literature had been reviewed [15–28, 31, 35–38], and a focus group discussion with four research experts (a nurse, a medical sociologist and two Jehovah’s Witnesses) was carried out. They discussed the list of questions regarding critical issues related to JWs’ stance on refusing blood transfusions and decided which issues to address. A preliminary questionnaire was pre-tested on ten nursing students via a communication platform used at the Poznan University of Medical Sciences for educational purposes (Microsoft Teams), which resulted in reformulating the three questions. It was then re-evaluated by the same experts: a nurse, a sociologist and two JWS.

The final version of the questionnaire consisted of 25 questions divided into four sections. The first dealt with students’ demographic data. The second section addressed students’ knowledge and awareness of JWs’ stance in refusing blood transfusions. The third section included questions about students’ opinions on the bioethical and legal dilemmas related to JWs’ stance in refusing blood transfusions. The last section referred to students’ educational needs regarding *bloodless medicine*, i.e., non-blood management strategies to minimise blood loss during surgery and obviate the need for blood transfusions (Supplementary material).

### Participants and setting

Nursing and midwifery master’s students were targeted for recruitment. The rationale behind choosing such students was two-fold: firstly, after completing the first stage of studies (3 years), which ends with a Bachelor’s Degree in Nursing or Midwifery, they are already qualified healthcare professionals and during the second stage (2 years), i.e. master’s studies, the vast majority already worked professionally in a variety of healthcare facilities; secondly, as qualified nurses and midwives who already worked in the profession, they were liable to face bioethical and legal dilemmas related to caring for a JW patient who refuses a blood transfusion in a life-threatening situation.

The inclusion criteria were: (1) being a nursing or midwifery master’s student, (2) being enrolled in the Poznań University of Medical Science (PUMS), (3) being willing to participate in the study, and (4) providing written informed consent before completing the survey.

### Data collection

The study was conducted between October and November 2023 among master’s students of nursing and midwifery at PUMS. Students were recruited during regular classes.

Before completing the survey, all students were informed by two members of the research team (JD and KG) about the study’s aim, as well as its voluntary, anonymous, confidential and non-compensatory character. They were also instructed about their right to abandon the survey without consequences. After informed consent was obtained from all students who agreed to complete the survey, all participants received a QR code and, once they had scanned it with their smartphones, they received access to the questionnaire posted on a Web platform. Completing the questionnaire took between 8 and 10 min.

### Ethical issues

This study followed the principles of the Declaration of Helsinki [44]. Ethics and research governance approval were also obtained from the Poznan University of Medical Sciences Bioethics Committee (KB – 760/22). All participants provided written informed consent before completing the survey.

### Data analysis

All analyses were conducted using the R Project for Statistical Computing [45], where we utilised various open-source R packages such as *tidyverse* [46], *flextable* [47] and *ggplot* [48] for tasks including data manipulation, statistical analysis and data visualisation.

We conducted a comprehensive analysis to examine potential statistical differences among the socio-demographic categories of students participating in the survey. Firstly, we implemented a descriptive analysis, offering insights into the variability and tendencies of the data. We also employed graphical representations of data, such as density curves, histograms and correlation plots, to depict the observed patterns visually. Finally, to rigorously assess the differences between various categories of survey participants, we applied formal statistical tests. A two-tail t-test for the mean and a chi-square test were employed to scrutinise the differences in variable distributions, ensuring a robust evaluation of the statistical significance of variations observed. Comparisons of 95% confidence intervals for the mean values were also undertaken to bolster the reliability of the findings. The analytical procedures chosen were paramount in providing a thorough and systematic exploration of the data, enabling a nuanced comprehension of potential distinctions between student groups and augmenting the scientific rigour of our study.

The main goal of our analysis was to assess the students’ knowledge of JWs’ stance in refusing blood transfusions. Respondents were presented with dozens of statements, some intentionally false, describing reasons for refusing an allogenic blood transfusion and medical procedures and the blood products accepted and those JWs would refuse. In total each respondent determined the truth of 51 sentences, based on which we built three indices of knowledge covering distinct aspects of JWs’ stance in refusing blood transfusions. While Index 1 measured the general knowledge of JWs’ stance in refusing blood transfusions; Index 2 measured knowledge regarding blood products approved by JWs; and Index 3 measured knowledge regarding medical procedures accepted by JWs. Note that each index consists of 17 statements formulated as *a priori* in a questionnaire to measure the students’ knowledge (consult Supplementary Materials for details). For each respondent the value of each index ranged from 0 (if none of the sentences were indicated correctly) to 17 (if the respondent indicated all the sentences correctly).

## Results

Of the 349 students approached, 302 (86.5%) participated in the study by completing the questionnaire (Table 1). Forty-seven students who refused to participate did so because they were either absent during the classes, lacked interest in the study or were unwilling to discuss their opinions. The feedback on surveys from the nursing students (NSs) was 145/188 (77.12%), and from the midwifery students (MSs) 157/161 (97.51%).

**Table 1:** Study participants

Characteristics	Total (*n* = 302)	Nursing (*n* = 145)	Midwifery (*n* = 157)	*p*-value
**Sex:**				
Woman	289 (95.7%)	134 (92.4%)	155 (98.7%)	
Man	13 (4.3%)	11 (7.6%)	2 (1.3%)	0.020^b)^
**Works professionally**:				
Yes	193 (63.9%)	125 (86.2%)	68 (43.3%)	
No	109 (36.1%)	20 (13.8%)	89 (56.7%)	< 0.001^b)^
*Seniority* [in months]	10.7 ± 1.7^a)^	9.4 ± 1.4^a)^	13.0 ± 4.2^a)^	0.120^c)^
**Importance of religion in life**:				
Significant	29 (9.6%)	11 (7.6%)	18 (11.5%)	
Rather big	70 (23.2%)	36 (24.8%)	34 (21.7%)	
Little	126 (41.7%)	58 (40.0%)	68 (43.3%)	
None	77 (25.5%)	40 (27.6%)	37 (23.6%)	0.540^b)^
**Earlier professional experience with a person who refused an allogeneic blood transfusion because of his or her religious beliefs**:				
Yes	57 (18.9%)	25 (17.2%)	32 (20.4%)	
No	245 (81.1%)	120 (82.8%)	125 (79.6%)	0.580^b)^

*Notes* (a) Mean value ± 95% Confidence Interval. (b) Chi-square test. (c) t-test for the mean

The sample comprised 145 NSs (48%) and 157 MSs (52%), all of Polish origin. While women predominated over men in the student body (95.7% vs. 4.3%), this disproportion results from the fact that both courses are strongly gendered in Poland. In 2021 women accounted for 73.76% of all medical and healthcare students in the country and this disproportion was even higher among nursing and midwifery students (89% and 99.54% respectively) [49].

Less than one-third (32.8%) of students claimed religion played any significant role in their life (32.4% NSs and 33.2% MSs) and 67.2% declared it was of little or no importance (67.6% NSs and 66.9% MSs).

A considerable number of respondents were professionally active (63.9%). The proportion of NSs working in their profession, however, was double that of MSs (86.2% vs. 43.3%, p *<* 0.001). 18.9% of respondents said that they had prior professional experience with patients who refused allogeneic blood transfusions because of their religious beliefs (17.2% and 20.4% MS).

**Fig. 1:**
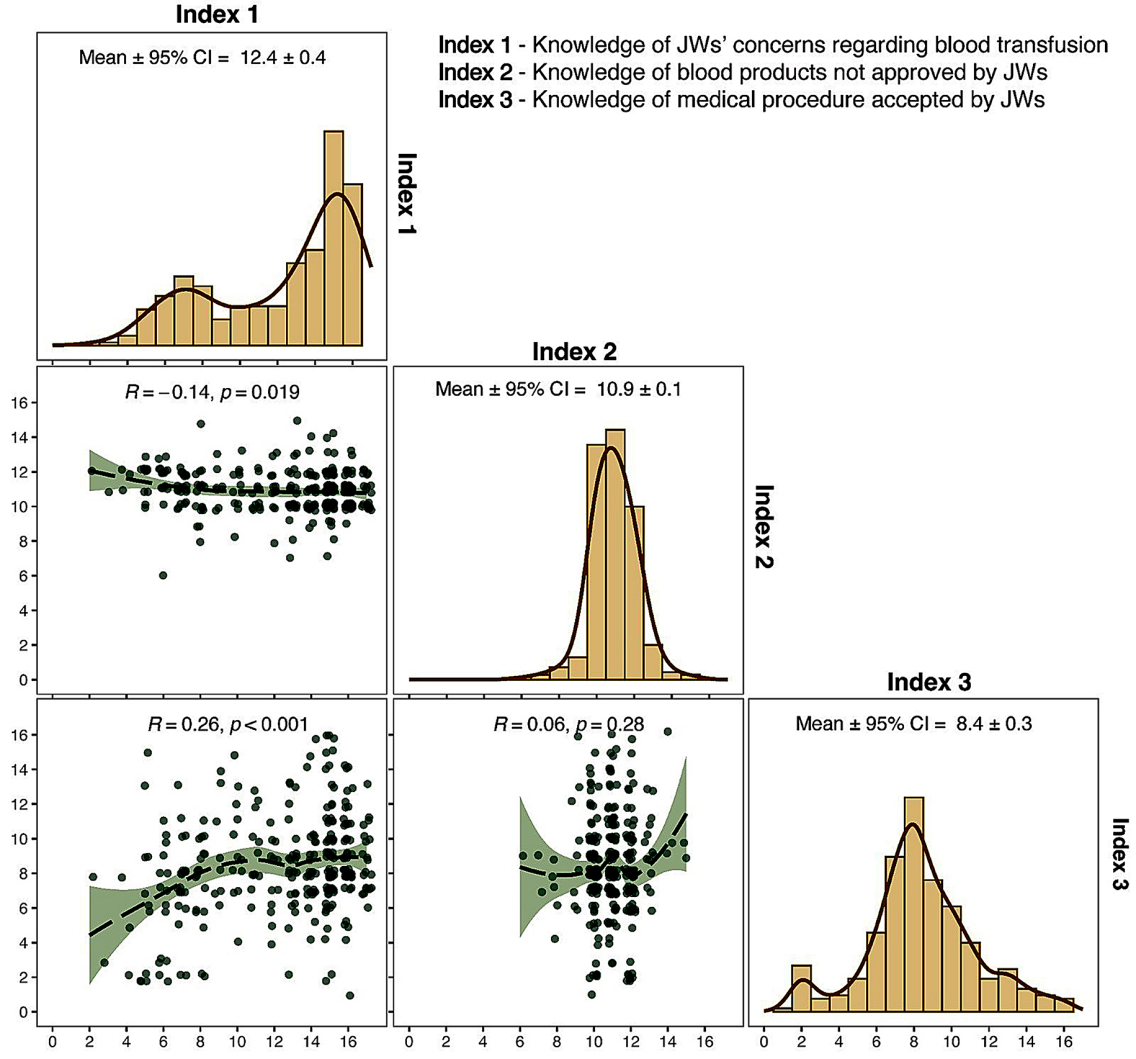
Histograms and correlation plots for indexes of students’ knowledge on JWs’ stand toward blood transfusions

Our analysis began by assessing the student’s knowledge of JWs’ stance in refusing blood transfusions. Figure 1 presents the distribution of the scores and correlation plots between the indices of students’ knowledge of JWs’ stance on blood transfusions. The results showed that the students scored highest on Index 1, with a mean score of 12.4, indicating that they had the greatest knowledge of JWs’ position on blood transfusion, on average correctly answering over 12 out of 17 statements. The highest mean score for Index 1 was followed by Index 2, with a mean score of 10.9, and Index 3, with the lowest mean score of 8.4, reflecting less knowledge about specific blood products and accepted medical procedures respectively. The correlations between all three indices were also generally low, highlighting the distinct nature of the domains of knowledge. In fact, Pearson’s linear correlation between Index 1 and Index 2 was − 0.14 (*p* = 0.019), indicating a significant, albeit only slightly negative relationship. The correlation between Index 1 and Index 3 was 0.26 (*p* < 0.001), indicating a significant but moderately positive relationship. The weakest Pearson correlation of 0.06 was between Index 2 and Index 3 (*p* = 0.28), indicating almost no relationship. In conclusion, while students have a good knowledge of the JW position on blood transfusion, their knowledge of specific blood products and accepted medical procedures is limited, suggesting the need for increased educational efforts to improve students’ overall understanding of medical practices accepted by the JW.

While the overall mean scores provide a general overview, specific group comparisons highlight nuanced differences. Table 2 compares the mean values of the indices measuring students’ knowledge of JWs’ stand on blood transfusions in groups delimited by selected socio-demographic characteristics.

**Table 2:** Students’ knowledge regarding JWs’ stand toward blood transfusions by socio-demographic characteristics

Characteristics	Index 1: Knowledge regarding JWs’ blood transfusion concerns [Mean ± 95 CI]	Index 2: Knowledge on blood products not approved by JWs [Mean ± 95 CI]	Index 3: Knowledge on medical procedures accepted by JWs [Mean ± 95 CI]
Total	12.4 ± 0.4	10.9 ± 0.1	8.4 ± 0.3
Nursing	12.1 ± 0.6	11.0 ± 0.2	9.3 ± 0.5
Midwifery	12.7 ± 0.6	10.8 ± 0.2	7.6 ± 0.4
p-value^a)^	0.190	0.310	**< 0.001**
Working: yes	12.3 ± 0.5	11.0 ± 0.2	8.8 ± 0.4
Working: no	12.7 ± 0.7	10.8 ± 0.2	7.6 ± 0.5
p-value^a)^	0.430	0.230	**< 0.001**
Very/rather significant role of religion	12.4 ± 0.7	10.9 ± 0.2	8.9 ± 0.6
Little/no role of religion	12.4 ± 0.5	10.9 ± 0.2	8.2 ± 0.4
p-value^a)^	0.960	0.880	**0.050**
Experienced refusal	12.8 ± 1.0	11.0 ± 0.3	8.9 ± 0.8
Not experienced refusal	12.3 ± 0.5	10.9 ± 0.2	8.3 ± 0.4
p-value^a)^	0.450	0.400	0.150

*Note* Statistically significant differences are written in boldface. a) t-test for the mean

The results show that midwifery students have greater knowledge regarding JWs’ concerns about blood transfusion but poorer knowledge of blood products and medical procedures accepted by JWs. The differences between the two categories of students are only significant, however, for the third index (the mean for nurses is 9.3, while for midwifery students it is 7.6, with *p* < 0.001), possibly indicating nurses’ deeper understanding of the issue of medical procedures. The participants’ employment status also plays a role, as those not currently working tended to have slightly lower mean scores (7.6 vs. 8.8, *p* < 0.001) in knowledge related to blood products and medical procedures accepted by JWs. Participants who attached little or no importance to religion and those who had never experienced a refusal also tended to have slightly lower knowledge scores in their awareness of medical the procedures accepted by JWs. The differences, however, remain negligible at *p* < 0.05.

**Fig. 2:**
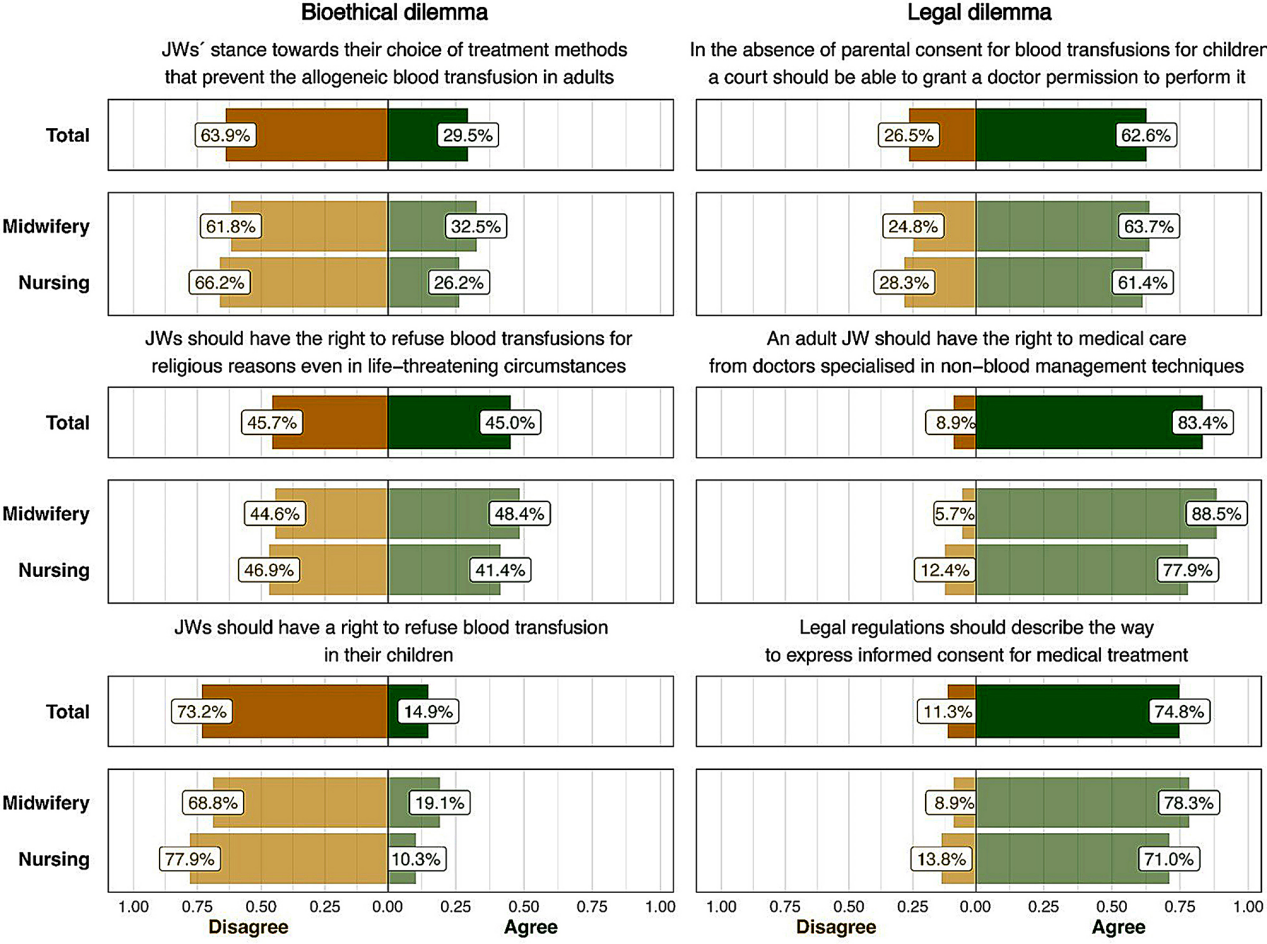
Students’ dilemmas related to JW’s stance toward blood transfusions

Figure 2 outlines students’ perception of the bioethical and legal dilemmas surrounding the refusal of blood transfusion in JW patients in total and broken down into two groups of students, i.e., MSs and NSs. The majority of students experience bioethical dilemmas as most of them disagree with the right of JW parents to refuse blood transfusion for JW children (73.2% overall, with nurses tending to agree more often than midwifery students: 77.9% vs. 68.8%) and showed a limited understanding of JWs’ position on their choice of treatment methods (63.9% in total: 66.2 for nursing students and 61.8 for midwifery students), as well as agreeing that JWs should have the right to refuse blood transfusions on religious grounds in life-threatening circumstances (45.7% in general, with nursing students more likely to agree than midwifery students: 46.9 vs. 44.6). Regarding legal dilemmas, most students (83.4%) agreed that adult JW patients should have access to medical care using non-blood management techniques (midwifery students were more likely to agree than nursing students: 88.5% vs. 77.9%). Respondents also felt that the guardianship court should authorise blood transfusions for JW children in cases where parental consent is withheld (62.6%, with 88.5% of midwifery students and 77.9% of nursing students agreeing). A clear majority (74.8%) of participants also agreed that an individual’s decision to refuse treatment should be subject to legal regulation, with midwifery students (78.3%) more likely to agree than nurses (71.0%).

Table 3 illustrates the variations in students’ views regarding bioethical and legal dilemmas across categories delineated by socio-demographic characteristics. Although there is no discernible trend in the influence of specific socio-demographics, some interesting differences were observed. The results demonstrate that the differences between survey participant groups are negligible in almost all cases, so we will briefly describe the differences between midwifery and nursing students. MSs agree more strongly than their nursing counterparts with JWs’ stance on treatment methods in which they refuse allogeneic blood transfusion in adults (32.5% vs. 26.2%). While a slightly higher proportion of MSs support the right of JWs to refuse blood transfusions in life-threatening circumstances, NSs display a marginally higher inclination toward disagreement (48.4% vs. 41.4%). A discrepancy exists in accepting legal regulations describing the way to express informed consent for medical treatment, with MSs registering a notably higher agreement percentage than their nursing counterparts (78.3% vs. 71.0%). The only significant discrepancy between students of nursing and midwifery surfaces in their acceptance of the right to medical care from doctors specialised in non-blood management techniques, with MSs registering a notably higher agreement percentage than their nursing counterparts (88.5% vs. 77.9%, *p* = 0.040). The results also show that the distribution of opinions on whether JWs should have the right to refuse blood transfusions on religious grounds, even in life-and-death situations, is firmly based on the perceived role of religion in their lives (*p* = 0.020).

**Table 3:** Students’ dilemmas related to JWs’ stance towards blood transfusion by socio-demographic characteristics

Question	Total		Nursing	Midwifery	Working yes	Working no	Very/rather significant role of religion	Little/none role of religion	Experienced refusal	Not experienced refusal
**Agree with JWs’ stance towards their choice of treatment methods that obviate allogeneic blood transfusion in adults:**										
Agree		89 (29.5%)	38 (26.2%)	51 (32.5%)	57 (29.5%)	32 (29.4%)	26 (26.3%)	63 (31.0%)	22 (38.6%)	67 (27.3%)
Disagree		193 (63.9%)	96 (66.2%)	97 (61.8%)	121 (62.7%)	72 (66.1%)	67 (67.7%)	126 (62.1%)	31 (54.4%)	162 (66.1%)
Do not know		20 (6.6%)	11 (7.6%)	9 (5.7%)	15 (7.8%)	5 (4.6%)	6 (6.1%)	14 (6.9%)	4 (7.0%)	16 (6.5%)
*Chi-square test*		-	χ2 = 1.63; df = 2; *p* = 0.440		χ2 = 1.19; df = 2; *p* = 0.550		χ2 = 0.91; df = 2; *p* = 0.630		χ2 = 3.00; df = 2; *p* = 0.220	
**JWs should have the right to refuse blood transfusions for religious reasons, even in life-or-death circumstances**:										
Agree	136 (45.0%)		60 (41.4%)	76 (48.4%)	86 (44.6%)	50 (45.9%)	34 (34.3%)	102 (50.2%)	27 (47.4%)	109 (44.5%)
Disagree	138 (45.7%)		68 (46.9%)	70 (44.6%)	86 (44.6%)	52 (47.7%)	56 (56.6%)	82 (40.4%)	25 (43.9%)	113 (46.1%)
Do not know	28 (9.3%)		17 (11.7%)	11 (7.0%)	21 (10.9%)	7 (6.4%)	9 (9.1%)	19 (9.4%)	5 (8.8%)	23 (9.4%)
*Chi-square test*	-		χ2 = 2.72; df = 2; *p* = 0.260		χ2 = 1.67; df = 2; *p* = 0.430		**χ2 = 7.55; df = 2;** *p* ** = 0.020**		χ2 = 0.16; df = 2; *p* = 0.930	
**JWs should have a right to refuse blood transfusion on behalf of their children**:										
Agree	45 (14.9%)		15 (10.3%)	30 (19.1%)	28 (14.5%)	17 (15.6%)	10 (10.1%)	35 (17.2%)	14 (24.6%)	31 (12.7%)
Disagree	221 (73.2%)		113 (77.9%)	108 (68.8%)	143 (74.1%)	78 (71.6%)	77 (77.8%)	144 (70.9%)	38 (66.7%)	183 (74.7%)
Do not know	36 (11.9%)		17 (11.7%)	19 (12.1%)	22 (11.4%)	14 (12.8%)	12 (12.1%)	24 (11.8%)	5 (8.8%)	31 (12.7%)
*Chi-square test*	-		χ2 = 4.75; df = 2; *p* = 0.090		χ2 = 0.24; df = 2; *p* = 0.890		χ2 = 2.71; df = 2; *p* = 0.260		χ2 = 5.39; df = 2; *p* = 0.070	
**In the absence of parental consent for blood transfusions for children, a court should be able to grant a doctor permission to perform one**:										
Agree	189 (62.6%)		89 (61.4%)	100 (63.7%)	124 (64.2%)	65 (59.6%)	62 (62.6%)	127 (62.6%)	43 (75.4%)	146 (59.6%)
Disagree	80 (26.5%)		41 (28.3%)	39 (24.8%)	49 (25.4%)	31 (28.4%)	25 (25.3%)	55 (27.1%)	9 (15.8%)	71 (29.0%)
Do not know	33 (10.9%)		15 (10.3%)	18 (11.5%)	20 (10.4%)	13 (11.9%)	12 (12.1%)	21 (10.3%)	5 (8.8%)	28 (11.4%)
*Chi-square test*	-		χ2 = 0.49; df = 2; *p* = 0.780		χ2 = 0.64; df = 2; *p* = 0.730		χ2 = 0.28; df = 2; *p* = 0.870		χ2 = 5.19; df = 2; *p* = 0.070	
**An adult JW should have the right to medical care from doctors specialised in non-blood management techniques**:										
Agree	252 (83.4%)		113 (77.9%)	139 (88.5%)	160 (82.9%)	92 (84.4%)	81 (81.8%)	171 (84.2%)	48 (84.2%)	204 (83.3%)
Disagree	27 (8.9%)		18 (12.4%)	9 (5.7%)	18 (9.3%)	9 (8.3%)	12 (12.1%)	15 (7.4%)	5 (8.8%)	22 (9.0%)
Do not know	23 (7.6%)		14 (9.7%)	9 (5.7%)	15 (7.8%)	8 (7.3%)	6 (6.1%)	17 (8.4%)	4 (7.0%)	19 (7.8%)
*Chi-square test*	-		χ2 = 6.30; df = 2; p = 0.040		χ2 = 0.13; df = 2; *p* = 0.940		χ2 = 2.18; df = 2; *p* = 0.340		χ2 = 0.04; df = 2; *p* = 0.980	
**Legal regulations should describe the way to express informed consent for medical treatment**:										
Agree	226 (74.8%)		103 (71.0%)	123 (78.3%)	146 (75.6%)	80 (73.4%)	79 (79.8%)	147 (72.4%)	47 (82.5%)	179 (73.1%)
Disagree	34 (11.3%)		20 (13.8%)	14 (8.9%)	20 (10.4%)	14 (12.8%)	10 (10.1%)	24 (11.8%)	4 (7.0%)	30 (12.2%)
Do not know	42 (13.9%)		22 (15.2%)	20 (12.7%)	27 (14.0%)	15 (13.8%)	10 (10.1%)	32 (15.8%)	6 (10.5%)	36 (14.7%)
*Chi-square test*	-		χ2 = 2.45; df = 2; *p* = 0.290		χ2 = 0.43; df = 2; *p* = 0.810		χ2 = 2.19; df = 2; *p* = 0.330		χ2 = 2.25; df = 2; *p* = 0.330	

Note Statistically significant differences are written in boldface

Among students for whom religion plays a very or fairly important role 34.3% agree that JWs should have this right. Conversely, among those who see religion as playing little or no role in their lives, 50.2% agree that JWs should have the right to refuse blood transfusions. Those who see religion as very important in their lives are therefore more likely to oppose the right to refuse transfusions, while those who see religion as less important are more likely to support this right.

Finally, Table 4 presents students’ educational needs regarding non-blood management techniques. The results indicate that many nurses and midwives have had no courses on non-blood management techniques (overall 61.3%, with significantly (*p* < 0.001) more midwifery students (75.2%) reporting having had no such courses during their studies, compared to nursing students (46.2%)). There was also a significant consensus in favour of the inclusion of mandatory courses on strategies to minimise blood loss in medical curricula (overall support above 80%, with midwifery students significantly more likely to agree (85.4%) than their nursing counterparts (75.2%), *p* < 0.001. A relatively small percentage of participants (11.3%) felt adequately prepared to care for patients who require non-blood management techniques despite this inclination, but nursing students were significantly (*p* < 0.001) more likely to report being prepared than midwifery students (17.9% vs. 5.1%; *p* < 0.01). The findings underscore the need for targeted educational interventions and training programmes to bridge gaps in healthcare professionals’ preparedness for non-blood management, especially given the apparent positive disposition toward such training courses. Note that the differences between the nursing and midwifery students are statistically significant, except for their willingness to expand their knowledge about non-blood management techniques. In both groups, the vast majority of students declared the intention to expand their knowledge. Note that the differences for other socio-demographic categories are insignificant.

**Table 4:** Students’ educational needs regarding non-blood management techniques by socio-demographic characteristics

Question	Total	Nursing	Midwifery	Working yes	Working no	Very/rather significant role of religion	Little/none role of religion	Experienced refusal	Not experienced refusal
**Declaration of having classes on non-blood management techniques that involve strategies of avoiding the use of blood transfusion and providing care to patients who refuse blood transfusion:**									
Yes	52 (17.2%)	39 (26.9%)	13 (8.3%)	39 (20.2%)	13 (11.9%)	19 (19.2%)	33 (16.3%)	8 (14.0%)	44 (18.0%)
No	185 (61.3%)	67 (46.2%)	118 (75.2%)	109 (56.5%)	76 (69.7%)	62 (62.6%)	123 (60.6%)	35 (61.4%)	150 (61.2%)
Do not know	65 (21.5%)	39 (26.9%)	26 (16.6%)	45 (23.3%)	20 (18.3%)	18 (18.2%)	47 (23.2%)	14 (24.6%)	51 (20.8%)
*Chi-square test*	-	χ2 = 29.23; df = 2; p < 0.001		χ2 = 5.57; df = 2; *p* = 0.060		χ2 = 1.14; df = 2; *p* = 0.560		χ2 = 0.72; df = 2; *p* = 0.700	
**Willingness to increase knowledge about non-blood management techniques**:									
Yes	272 (90.1%)	125 (86.2%)	147 (93.6%)	171 (88.6%)	101 (92.7%)	90 (90.9%)	182 (89.7%)	51 (89.5%)	221 (90.2%)
No	22 (7.3%)	14 (9.7%)	8 (5.1%)	16 (8.3%)	6 (5.5%)	8 (8.1%)	14 (6.9%)	4 (7.0%)	18 (7.3%)
Do not know	8 (2.6%)	6 (4.1%)	2 (1.3%)	6 (3.1%)	2 (1.8%)	1 (1.0%)	7 (3.4%)	2 (3.5%)	6 (2.4%)
*Chi-square test*	-	χ2 = 4.95; df = 2; *p* = 0.080		χ2 = 1.30; df = 2; *p* = 0.520		χ2 = 1.63; df = 2; *p* = 0.440		χ2 = 0.21; df = 2; *p* = 0.900	
**Courses on strategies to minimise blood loss during surgery and prevention of blood transfusion in medical curricula should be mandatory**:									
Yes	243 (80.5%)	109 (75.2%)	134 (85.4%)	152 (78.8%)	91 (83.5%)	81 (81.8%)	162 (79.8%)	47 (82.5%)	196 (80.0%)
No	34 (11.3%)	13 (9.0%)	21 (13.4%)	20 (10.4%)	14 (12.8%)	10 (10.1%)	24 (11.8%)	9 (15.8%)	25 (10.2%)
Do not know	25 (8.3%)	23 (15.9%)	2 (1.3%)	21 (10.9%)	4 (3.7%)	8 (8.1%)	17 (8.4%)	1 (1.8%)	24 (9.8%)
*Chi-square test*	-	χ2 = 21.65; df = 2; p < 0.001		χ2 = 4.95; df = 2; *p* = 0.080		χ2 = 0.22; df = 2; *p* = 0.900		χ2 = 4.93; df = 2; *p* = 0.090	
**Being prepared to care for a patient who requires treatment with non-blood management techniques**									
Yes	34 (11.3%)	26 (17.9%)	8 (5.1%)	25 (13.0%)	9 (8.3%)	11 (11.1%)	23 (11.3%)	10 (17.5%)	24 (9.8%)
No	242 (80.1%)	100 (69.0%)	142 (90.4%)	149 (77.2%)	93 (85.3%)	82 (82.8%)	160 (78.8%)	42 (73.7%)	200 (81.6%)
Do not know	26 (8.6%)	19 (13.1%)	7 (4.5%)	19 (9.8%)	7 (6.4%)	6 (6.1%)	20 (9.9%)	5 (8.8%)	21 (8.6%)
*Chi-square test*	-	χ2 = 21.91; df = 2; p < 0.001		χ2 = 2.89; df = 2; *p* = 0.240		χ2 = 1.25; df = 2; *p* = 0.540		χ2 = 2.83; df = 2; *p* = 0.240	

Note Statistically significant differences are written in boldface

## Discussion

Poland remains one of Europe’s most ethnically and culturally homogeneous and religious countries. It has an extremely low rate of people of non-Polish descent, and Polish society is predominantly Christian. Most Poles identify as Roman Catholics (71.3%) [14]. Over the past few decades, however, Polish society has become more diverse. Demographic changes in Europe require that all healthcare professionals, including nurses and midwives, develop the knowledge and skills needed to provide holistic, patient-centred, culturally sensitive care [5, 6] and the growing body of literature in Poland stresses the importance of cultural competency in healthcare [50–57]. While many educational programmes that seek to develop nurses’ cultural competence have been implemented in Europe and elsewhere [58–64], this has only recently begun in Poland [65, 66].

Earlier studies have shown that the Polish public is relatively poorly informed about other cultures and religions. On the other hand, although JWs are more familiar than other faith groups, such as Muslims, Jews, Hindus and Buddhists, and 60% of people claim to know a JW personally, many Poles are still critical of JWs [67, 68]. More importantly, research has demonstrated that 61.3% of nurses in Poland have prior experiences with a patient with a distinct cultural background, less than half had heard the term *cultural competences* (47.2%), and 92.5% felt unprepared to care for patients from different cultures. 91.5% of nurses also declared that all nurses should know other cultures, including their impact on healthcare and disease (57.5%), be able to identify problems arising from cultural differences (59.4%) and have the skills required to overcome ethnocentrism, stereotypes and prejudices (59.4%) [54].

In another study 86.8% of nurses claimed to have had little or no contact with patients from a different culture or religion and 62.3% experienced difficulties interacting with such patients due to a lack of knowledge or communication skills. Finally, 74.3% of nurses admitted to having various stereotypes of Muslims, JWs, the Roma or Hindus and 55.7% had an unfavourable image of such patients [68]. A recent study by Zalewska-Puchała et al. showed that, since many Polish nurses revealed varying levels of social distance towards followers of various religions, there is a need to train nurses in transcultural nursing [69]. Walkowska et al., however, demonstrated that cross-cultural education increases future healthcare professionals’ levels of cultural competence and professional confidence [66].

This research therefore reports three significant findings. Firstly, it shows that future nurses and midwives have limited knowledge regarding JWs’ stance in refusing blood transfusions. Nursing students taking part in this study showed some general knowledge regarding JWs’ refusal of blood transfusion, but their awareness of blood products and medical procedures approved by JWs was relatively low. This result aligns with a previous study, indicating that while many nurses in Poland lack the cultural competences required to care for JW patients and, even though they tend to support adult JWs’ right to refuse a blood transfusion, they show little understanding of such a decision and expressed resentment towards JWs’ stance [42]. More than 83% of nurses in Lublin, Poland, claimed to have had contact with JW patients and more than half (50.02%) rejected JWs’ position concerning blood treatment, 44.23% admitting to having tried to persuade JW parents to change their minds and accept blood transfusions [70]. While 83% of anaesthesiologists, physicians and surgeons in France did not oppose the medical care of JWs, they remained committed to their primary focus: to save the patient, as long as it is not an end-of-life situation, and 67% admitted that in life and death situations, where there is a lack of alternative procedures, blood products should be administered [71]. Although German doctors stressed the importance of personal autonomy, they also referred to doctors’ consciences and their ethical professional obligations [25].

Secondly, this research also found that future nurses and midwives are aware of the bioethical and legal dilemmas healthcare professionals face when caring for JW patients. The majority, however, showed limited support for both JWs’ stance in their refusal of blood transfusions and their preferences for bloodless medicine. Less than half of respondents supported JWs’ right to refuse blood transfusions for religious reasons in life-threatening situations and the majority stressed JWs’ right to alternative, non-blood management techniques. Even fewer supported JW parents’ right to refuse blood transfusion for their children. Similar results were found in other studies, suggesting that in the case of infant or juvenile patients, blood transfusions should be performed even against parents’ will [69, 70].

Thirdly, these findings underscore the educational needs regarding cultural competences in nursing, both in terms of general knowledge regarding JWs’ stance in refusing blood transfusions and non-blood management techniques. Since nursing and midwifery students felt unprepared to care for JW patients, this study shows an urgent need to include transcultural nursing and strategies to minimise blood loss modules in university curricula and postgraduate nursing training [58–66].

## Limitations

This study has some limitations that should be acknowledged. Firstly, although the response rate was high (86.5%), the sample size was still small. Secondly, since 47 students decided against participating, this survey study solely represents the opinions of students who completed the questionnaire. Thirdly, nursing and midwifery students from only one Polish medical university participated in this study. For all these reasons, our results cannot be extrapolated to include the entire population of nursing and midwifery students, either in Poznan or Poland and further in-depth studies are required. Fourthly, it would be desirable to compare our findings with students of other departments and those in contact with patients who refuse blood transfusion, i.e. medicine or medical rescue. The questionnaire used in this survey was also ad hoc and, though we consulted four specialists in nursing, sociology and the culture of Jehovah’s Witnesses, it was not validated. Finally, this study is based exclusively on the quantitative method. Further in-depth studies based on qualitative methods are recommended in order better to understand students’ attitudes towards and experiences in providing medical care for JW patients.

Despite these limitations, there are some advantages to this study. Most importantly, as there is a scarcity of previous work on the topic, this research helps bridge the gap in research on the knowledge of future healthcare professionals on JWs’ stance toward blood transfusion. This study compares the knowledge of nursing and midwifery students and may also stimulate further discussion on the need for better education and increasing cross-cultural competences among future nurses and midwives, whose roles in caring for JW patients is vital.

## Conclusions

This study demonstrates that nursing and midwifery students possess inadequate knowledge regarding JWs’ stance on blood transfusions and their acceptance of specific blood products and medical procedures. It also shows that, despite being cognisant of the ethical and legal dilemmas of caring for JW patients, future nurses and midwives show limited support for patients’ autonomy to reject blood transfusions and their preferences for bloodless medicine. Finally, students articulated educational needs regarding cultural competencies on the JWs’ beliefs regarding blood transfusions and non-blood management techniques. Since culturally competent nurses and midwives must establish trust and approach all patients with respect for their cultural identity and values, this study reveals an urgent need to train future nurses and midwives in transcultural nursing and increase their cultural competencies. To achieve this goal, all medical curricula should include a transcultural nursing module akin to those in other European countries. Students should also be trained in the ways cultural norms and healthcare professionals’ personal beliefs may affect their decision-making, hinder patient communication and prevent individuals from receiving patient-centred and culturally sensitive care. Finally, future nurses and midwives must be taught and trained about the challenges of caring for JW patients, including ethical and legal dilemmas.

### Electronic supplementary material

Below is the link to the electronic supplementary material.

Supplementary Material 1

## Data Availability

Data generated as part of this study with replication codes for all analyses are available from the corresponding author upon reasonable request.

## Abbreviations

JW: Jehovah’s Witnesses
MSs: Midwifery students
NSs: Nursing students
