# Supplementary material for: Cultural competences among future nurses and midwives: a case of attitudes toward Jehovah’s witnesses’ stance on blood transfusion
Source: BMC Med Educ. 2024 Jun 15;24:663. doi: 10.1186/s12909-024-05646-1 (PMC11180393; doi:10.1186/s12909-024-05646-1)
Supplement: Supplementary file 1 — Supplementary Material 1 [file 12909_2024_5646_MOESM1_ESM.docx]

**Supplementary material. Supplementary material. Questionnaire**

Dear Madam! Dear Sir!

My name is Jan Domaradzki and I work at the Medical University of Poznań, where, together with my research team, I conduct research on the knowledge and knowledge and attitudes of nursing and midwifery students toward Jehovah’s Witnesses’ stance on blood transfusion.

We would like to invite you to share your opinion on this important topic. This survey should only take approximately 10-150 minutes of your time.

We would like to assure you that the research is completely anonymous and confidential (please do not sign it). As all responses are anonymous they cannot be traced back to the respondent. Moreover, while no personally identifiable information is captured your responses will be combined with those of many others and summarized in a report to further protect your anonymity. All information gathered will be used only for scientific purposes.

Please answer all questions in the questionnaire. Unless otherwise stated, please select only one answer. If you are not sure of the answer, please choose the one that seems most appropriate to you.

We would like to thank you in advance for your help, time and assistance in carrying out the study.

For any questions, or if you need assistance to complete this questionnaire, please contact:

Jan Domaradzki, dr. hab. n. hum.

Laboratory of Health Sociology and Social Pathology, Department of Social Sciences and Humanities

Poznan University of Medical Sciences

Rokietnicka 7, Poznań, Poland

tel./fax: 61 8452 770; e-mail: [jandomar@ump.edu.pl](mailto:jandomar@ump.edu.pl)

Your feedback is very important. We appreciate your time and help

Sincerely

Jan Domaradzki

**INFORED CONSENT FORM:**

1. I was informed by the Project Manager, Dr. hab. Jan Domaradzki about the planned scientific research, in particular about its assumptions, goals, course and method of conducting the research.

2. I understand all information provided to me regarding this research study.

3. I have been informed that participation in the research study is completely voluntary.

4. I have been informed that I can withdraw from participation in this research study at any time, without giving reasons, and my decision will not result in any penalties.

5. I have been informed that if I have any questions or doubts during the research study, I can contact the persons indicated in the information.

6. I voluntarily consent to participate in the study.

 yes  no

*1. Sex*

 Man

 Woman

*2. Faculty*

 nursing

 midwifery

*3. Do you already work as a healthcare professional?*

yes

no

*4. Do you already work as a healthcare professional?*

yes

no

*5. Seniority (in years)*

…………………

*6. What role does religion play in your life?*

 Significant, it influences my life decisions and choices

 Rather big, I try to follow religious principle in my life

 Little, I separate religion from public issues

 None, it is irrelevant to me

7. Have you ever had earlier professional experience with a person who refused an allogeneic blood transfusion because of his or her religious beliefs?

yes

no

*8. Jehovah’s witnesses’ refuse allogeneic blood transfusion for*

health/medical reasons

religious reasons

both medical and religious reasons

I do not know

*9. Jehovah’s witnesses’ refuse allogeneic blood transfusion because*

they refer to the biblical prohibition ingesting blood

they believe that blood is the seat of the soul

they believe that blood is impure

they argue that allogeneic blood transfusion can cause transfusion-transmitted infections and diseases

they argue that allogeneic blood transfusion can cause death

they do not trust in the healthcare system

they do not use the healthcare system

I do not know

*10. Jehovah’s witnesses’ are the only group that refuses blood transfusion*

yes

no

I do not know

11. Jehovah’s Witnesses’ prohibition of blood transfusion applies to:

infants before the first week of life

children

pregnant women

people above 75 years of age

people with disabilities

people on dialysis

people with blood disease, i.e. haemophilia

people who have to take immunoglobulins

all Jehovah’s Witnesses without any exception in reference to age, sex or health

12. Jehovah’s Witnesses’ refusal of blood transfusion concerns:

pre-operative autologous blood donation for re-infusion

taking white blood cells

acute normovolemic haemodilution

dialysis

extracorporeal circulation

intra-operative blood recovery

all answers are true

none of the above

I do not know

13. Jehovah’s Witnesses’ refusal of allogeneic blood transfusion concerns:

red blood cells

white blood cells

platelets

fractions from red blood cells

fractions from white blood cells

platelet-derived fractions

plasma-derived fractions

all answers are true

none of the above

I do not know

14. Jehovah’s Witnesses accept:

laboratory tests of autologous blood

laboratory tests of allogeneic blood

administration of local anaesthesia

administration of general anaesthesia

protective vaccinations

bone marrow transplant

orthopaedic procedures

surgical procedures involving autologous blood

organ transplantation involving autologous blood

pre-operative autologous blood donation for re-infusion

organ donation

plasmapheresis

cell labelling

using epidural blood patch

using of autologous platelet-rich gel

stem cell transplant

honorary blood donation

*15. I support with the Jehovah’s Witnesses’ stance towards their choice of treatment methods that prevent the allogeneic blood transfusion in adults*

 definitely yes

 possibly yes

 possibly no

 definitely no

 I do not know

*16. An adult Jehovah’s Witnesses should have the right to refuse blood transfusions for religious reasons even in life-threatening circumstances.*

 definitely yes

 possibly yes

 possibly no

 definitely no

 I do not know

*17. Jehovah’s Witnesses should have the right to refuse blood transfusions on behalf of their children.*

 definitely yes

 possibly yes

 possibly no

 definitely no

 I do not know

*18. In the absence of parental consent for blood transfusions for Jehovah’s Witness children a guardianship court should be able to grant a doctor permission to perform it.*

 definitely yes

 possibly yes

 possibly no

 definitely no

 I do not know

*19. An adult Jehovah’s Witnesses should have the right to medical care from doctors specialized in non-blood management techniques.*

 definitely yes

 possibly yes

 possibly no

 definitely no

 I do not know

*20. Legal regulations should describe the way to express informed consent for medical treatment, including the choice or refusal of any particular therapy*

 definitely yes

 possibly yes

 possibly no

 definitely no

 I do not know

*21.* Did you have any classes on non-blood management techniques (bloodless medicine) that involve strategies for avoiding blood transfusion and providing care to patients who refuse a blood transfusion?

 yes

 no

 I do not know

*22.* Would you like to extend your knowledge regarding non-blood management techniques?

 yes

 no

 I do not know

*23.* Do you think there should be a mandatory course on strategies to minimise blood loss during surgery and prevent blood transfusion (patient blood management and non-blood management techniques) in medical curricula?

 yes

 no

 I do not know

24. Do you feel prepared to care for a patient who requires treatment with non-blood management techniques?

 yes

 no

 I do not know
